# Direct local solvent probing by transient infrared spectroscopy reveals the mechanism of hydrogen-bond induced nonradiative deactivation†
†Electronic supplementary information (ESI) available: Experimental details, basic photophysics of **ADA**, transient electronic absorption, additional steady-state and transient IR spectra. See DOI: 10.1039/c7sc00437k



**DOI:** 10.1039/c7sc00437k

**Published:** 2017-05-16

**Authors:** Bogdan Dereka, Eric Vauthey

**Affiliations:** a Department of Physical Chemistry , University of Geneva , 30 Quai Ernest-Ansermet , CH-1211 Geneva 4 , Switzerland . Email: Eric.Vauthey@unige.ch

## Abstract

The fluorescence quenching of organic dyes *via* H-bonding interactions is a well-known phenomenon. However, the mechanism of this Hydrogen-Bond Induced Nonradiative Deactivation (HBIND) is not understood. Insight into this process is obtained by probing in the infrared the O–H stretching vibration of the solvent after electronic excitation of a dye with H-bond accepting cyano groups. The fluorescence lifetime of this dye was previously found to decrease from 1.5 ns to 110 ps when going from an aprotic solvent to the strongly protic hexafluoroisopropanol (**HFP**). Prompt strengthening of the H-bond with the dye was identified by the presence of a broad positive O–H band of **HFP**, located at lower frequency than the O–H band of the pure solvent. Further strengthening occurs within a few picoseconds before the excited H-bonded complex decays to the ground state in 110 ps. The latter process is accompanied by the dissipation of energy from the dye to the solvent and the rise of a characteristic hot solvent band in the transient spectrum. Polarization-resolved measurements evidence a collinear alignment of the nitrile and hydroxyl groups in the H-bonded complex, which persists during the whole excited-state lifetime. Measurements in other fluorinated alcohols and in chloroform/**HFP** mixtures reveal that the HBIND efficiency depends not only on the strength of the H-bond interactions between the dye and the solvent but also on the ability of the solvent to form an extended H-bond network. The HBIND process can be viewed as an enhanced internal conversion of an excited complex consisting of the dye molecule connected to a large H-bond network.

## Abstract 2

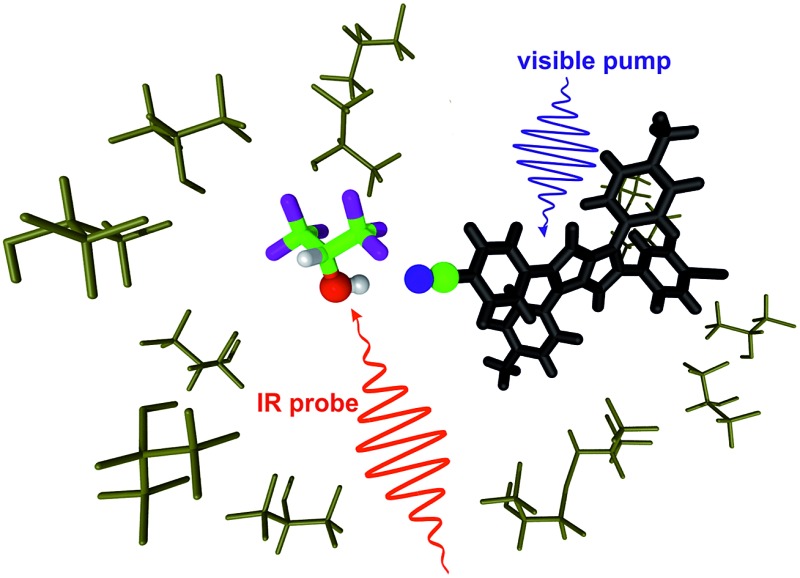
The hydrogen-bond induced quenching of an excited chromophore is visualised by probing O–H vibrations of the interacting solvent molecules.

## Introduction

**Chart 1:**
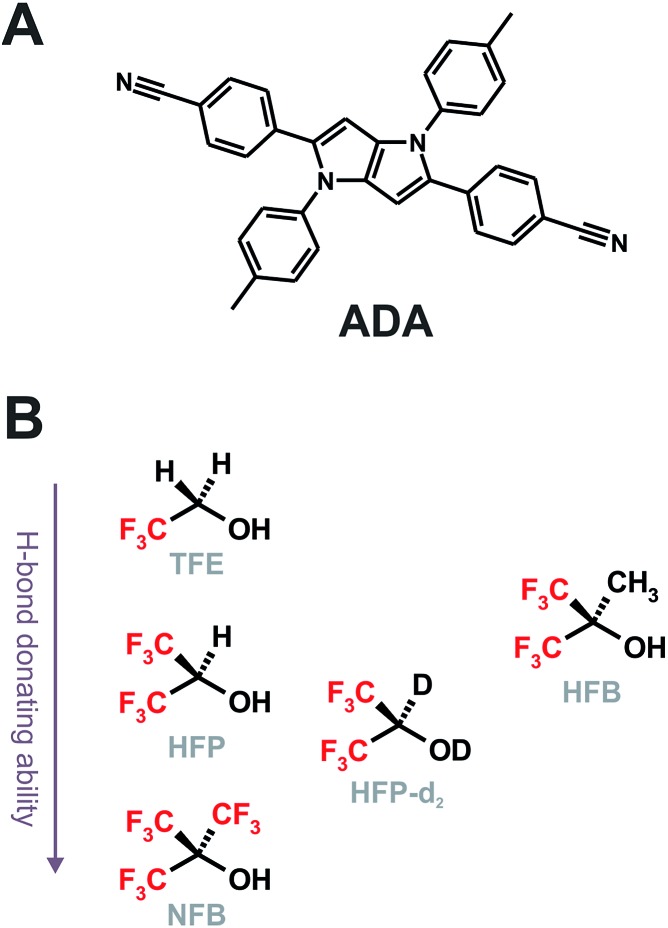
Structure of **ADA** (A) and of the superprotic solvents arranged according to their H-bond donating ability and structural similarity (B).

Hydrogen-bonding interactions play crucial roles in chemistry and biology.^1–3^ In photochemistry, H-bonds are usually involved in excited-state proton transfer and proton-coupled electron transfer reactions.^4–12^ In several cases, the quenching of a molecule in the electronic excited state can occur *via* H-bonding interactions but without the occurrence of the two afore-mentioned processes.^13–24^ The exact mechanism of this Hydrogen-Bond Induced Nonradiative Deactivation (HBIND) is not really understood. It has been observed in protic solvents with chromophores characterized by (i) the presence of H-bond accepting sites, such as carbonyl or nitro groups and (ii) a substantial increase of the charge-transfer character upon excitation leading to an increase of the electronic density on the H-bond accepting atom and, thus, to a strengthening of the H-bond with the solvent. The HBIND process manifests by a decrease of the excited-state lifetime with increasing the H-bond donating strength of the solvent,^16,18,21,23,24^ quantified by the Kamlet–Taft parameter *α*.^25,26^ For example, the fluorescence lifetime of eosin B drops from a few ns in aprotic solvents (*α* = 0) to 210 ps in butanol (*α* = 0.84) and to a few ps in water (*α* = 1.17).^21^ It should however be noted that solvents with a *α* parameter larger than that of water were not investigated. Recently, HBIND was found to be operative with a symmetric quadrupolar A–π–D–π–A molecule (**ADA**, Chart 1A),^27,28^ consisting of an electron-rich pyrrolo[3,2-*b*]pyrrole electron-donating core, D, with cyanophenyl acceptors (A) at positions 2 and 5.^29^ By monitoring the C≡N stretching frequency of **ADA** using time-resolved IR (TRIR) spectroscopy, it was shown that, whereas the S_1_ state is fully symmetric in non-polar solvents, a symmetry breaking of the electronic distribution is taking place in polar solvents. This process was identified by the presence of two distinct C≡N bands in the excited-state absorption spectrum, instead of one in non-polar solvents. The frequency splitting of these two bands, reflecting the extent of asymmetry, was found to increase with solvent polarity. Excited-state symmetry breaking results in a S_1_ state with the two C≡N ends having an uneven electronic density and, thus, a different basicity. In protic solvents with *α* < 1.3, further symmetry breaking due to the formation of H bonds of different strength between the solvent and the C≡N groups was found to take place on the timescale of diffusive solvation. However, no reduction of the excited-state lifetime was observed. On the other hand, in ‘superprotic’ solvents with *α* > 1.3, the lifetime was found to drop from ∼1.5 ns to 300 ps in trifluoroethanol (**TFE**, *α* = 1.51) and to 110 ps in hexafluoroisopropanol (**HFP**, *α* = 1.96), pointing to the occurrence of HBIND. Surprisingly, in nonafluoro-*tert*-butanol (**NFB**), the strongest H-bond donating solvent known (*α* > 2),^30^ the excited-state lifetime amounted to 950 ps, pointing to less efficient HBIND. The excited-state IR absorption spectrum of **ADA** in these superprotic solvents is characterized by two strongly split C≡N bands that were assigned to an asymmetric tight H-bonded complex.

The observation of such a complex makes **ADA** a valuable candidate to obtain deeper insight into the HBIND mechanism. HBIND should be a quite widespread process for organic dyes in aqueous solutions and thus proper understanding of its mechanism is not only important for our basic comprehension of photochemistry but should be highly beneficial to the development of new water-soluble fluorescent probes.

We report here on our investigation on the mechanism of the HBIND process with **ADA** using femtosecond TRIR spectroscopy. Here, contrary to our previous studies on the symmetry-breaking dynamics,^29,31^ we perform ‘solute-pump/solvent-probe’ TRIR measurements, namely we probe the temporal response of the O–H stretching mode of the solvent upon photoexcitation of **ADA** in **HFP**, perdeuterated **HFP** (**HFP-d_2_**) and in hexafluoro-*tert*-butanol (**HFB**, Chart 1B). We will mostly focus on **HFP**, since this is the solvent where HBIND is the most efficient. On the other hand, **HFP-d_2_** and **HFB** were selected because of their structural similarity to **HFP** and **NFB**, respectively. Their *α* parameter is expected to be slightly higher and lower than that of these two solvents.

The idea to monitor spectator vibrational modes of the solvent to obtain deeper insight into the excited-state dynamics of a solute molecule was first demonstrated by Hochstrasser and co-workers,^32,33^ and was recently applied by two other groups.^34–36^ This approach exploits the dependence of the shape, intensity and/or frequency of IR absorption bands of various vibrations to the local electric field or the temperature. It was used to monitor local field changes around a photoexcited molecule,^32,34^ or to follow the temperature rise of the solvent molecules around an excited molecule or a nanoparticle due to the heat released upon its chemical transformation,^35^ or non-radiative decay.^33,36^ Our approach is significantly different in the sense that the O–H stretching mode that we monitor is no longer a spectator mode when the solvent molecule is directly H-bonded to the solute. Additionally, it is a local vibrational mode whose frequency reports on the H-bond strength and it is spectrally well separated from the other solute and solvent vibrations. We will show that this strategy allows visualizing both the strengthening of the H-bond between the excited dye and the solvent and the ensuing non-radiative decay.

## Results

### **ADA** in **HFP** and acetone

**Fig. 1:**
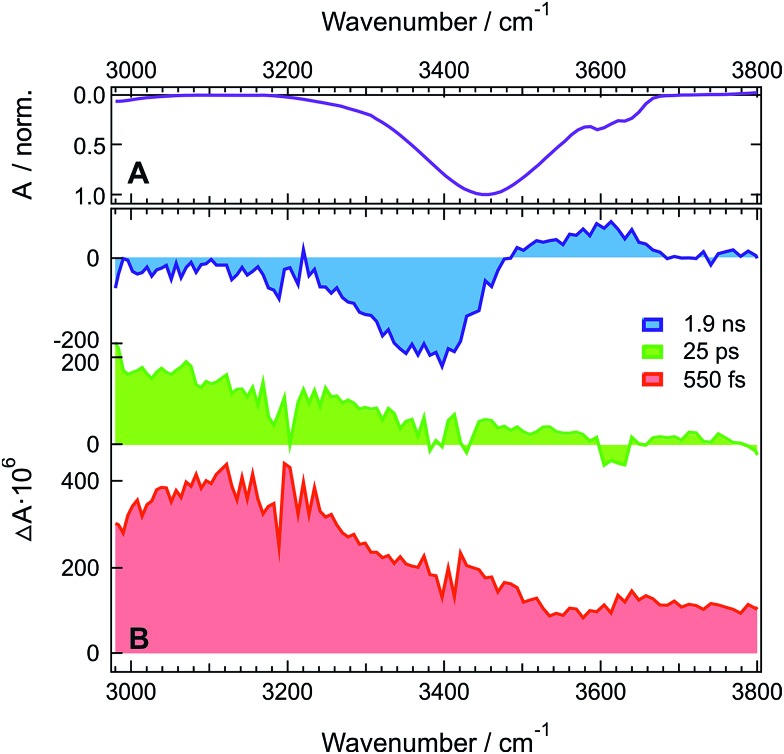
(A) Inverted stationary IR absorption spectrum of **HFP** in the O–H stretching region. (B) Transient IR absorption spectra recorded at three representative time delays after 400 nm excitation of **ADA** in **HFP**.

Fig. 1A shows the stationary IR absorption spectrum of pure **HFP** in the 3000–3800 cm^–1^ region. Whereas the broad absorption band at ∼3450 cm^–1^ corresponds to O–H stretching of the H-bonded oscillators and is a quite general feature of alcohols, the two distinct sharp peaks at 3596 and 3631 cm^–1^ are due to the free, non H-bonded, O–H stretching of **HFP**.^37–39^ The presence of these two distinct free OH bands in bulk **HFP** is in sharp contrast with the spectra of other alcohols for which the free OH band can only be observed upon significant dilution with an inert solvent and no conformational substructure is usually visible. TRIR spectra recorded at various time delays after excitation of **ADA** in **HFP** at 400 nm, close to maximum of the S_1_ ← S_0_ absorption band (Fig. S1†), are shown in Fig. 1B. The temporal evolution of the transient absorption over the whole spectral window was analysed globally,^40,41^ assuming a series of three successive exponential steps (A → B → C →) with 4.2, 110 ps and ≫10 ns time constants. The resulting evolution-associated difference spectra (EADS, Fig. S5A†) are very similar to the three transient spectra shown in Fig. 1B.

The early transient spectrum (red in Fig. 1, EADS A in Fig. S5A†) consists of a broad positive feature covering the whole 3000–3800 cm^–1^ spectral window with a maximum between 3100 and 3200 cm^–1^. This spectrum evolves in 4.2 ps to a spectrum (green in Fig. 1, EADS B in Fig. S5A†) with weaker amplitude and with a maximum shifted to lower frequencies. Finally, this spectrum transforms in 110 ps to another one (blue in Fig. 1, EADS C in Fig. S5A†) dominated by a negative band at ∼3400 cm^–1^ and a weaker positive band around 3600 cm^–1^. This final spectrum remains constant up to 2 ns, the longest time delay of the experiment.

**Fig. 2:**
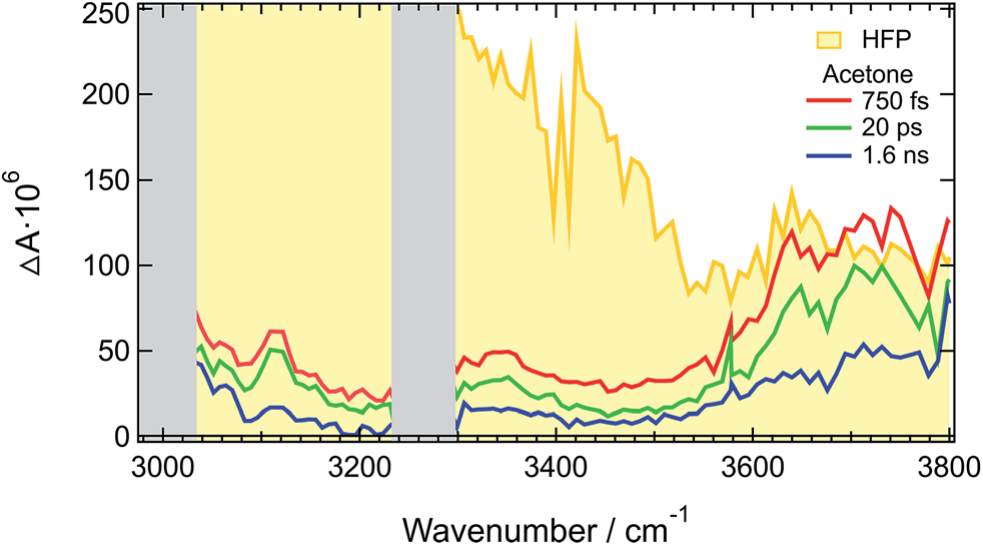
Transient IR absorption spectra recorded at three representative time delays after 400 nm excitation of **ADA** in acetone. An early transient spectrum measured in **HFP** using the same experimental conditions is shown for comparison.

TRIR spectra recorded under the same experimental conditions in the aprotic acetone show only the weak transient band above 3500 cm^–1^ that decays with the same lifetime, 1.6 ns, as the S_1_ state of **ADA** in this solvent (Fig. 2 and S5B†). This indicates that this feature originates from **ADA** in the excited state, whereas the transient absorption measured in **HFP** below 3500 cm^–1^ is due to the O–H stretching vibration of the solvent. By comparing the one- and two-photon absorption spectra of **ADA**, the S_2_ ← S_1_ transition can be estimated to be around 4000 cm^–1^ and to be one-photon allowed, in agreement with the selection rules for centrosymmetric molecules.^28,42^ Therefore, the transient feature above 3500 cm^–1^ can be assigned to the onset of the S_2_ ← S_1_ absorption band of **ADA**. In **HFP**, this band decays apparently faster than the S_1_ state of **ADA**. However, the transient electronic absorption spectra in this solvent (Fig. S4†) point to a drastic blue shift of the S_*n*_ ← S_1_ absorption band during the first few tens of picoseconds due to the stabilization of the S_1_ state upon tight H-bond formation. The same process occurs here with the S_2_ ← S_1_ band leading to its rapid shift out of the spectral window. The TRIR spectra in acetone exhibit additionally two weak positive bands at 3115 cm^–1^ and 3345 cm^–1^ that are assigned to aromatic C–H vibrations of **ADA** in the S_1_ state.

The presence of a transient O–H stretching band at the earliest time delays in **HFP** (Fig. 1B) points to a prompt response of this solvent mode to the electronic excitation of **ADA**. This can be explained by a strong coupling between **ADA** and the **HFP** molecules contributing to the signal, such as that associated with an H-bonded complex. The frequency upshift of the C≡N stretch band observed in the stationary IR spectrum of **ADA** upon increasing the H-bond donating strength of the solvent (*vide infra*) indicates that H-bond interactions are already operative in the ground state.^43–45^ Optical excitation of **ADA**, which results in an increase of electronic density at the cyano ends, leads to a concurrent strengthening of the H-bond interactions and, thus, to a decrease of the O–H stretching frequency of the interacting **HFP** molecules. In the TRIR spectrum, this should appear as a negative band around 3400 cm^–1^ due to the depletion of more weakly H-bonded **HFP** molecules, and as a positive band at lower frequency arising from the more strongly bonded **HFP** molecules. The early spectrum (red in Fig. 1B, EADS A in Fig. S5A†) are in only partial agreement with such expectations, with the broad positive band around 3100 cm^–1^ that can be ascribed to the **HFP** molecules H-bonded to **ADA** in the S_1_ state. However, no bleach feature due to the disappearance of more weakly bonded **HFP** molecules can be observed. This absence can be explained by a substantial increase of the transition dipole moment of the O–H vibration with increasing H-bond strength.^46–48^ This vibrational non-Condon effect has been shown to be responsible for an increase of the transition dipole by a factor 1.7 with decreasing frequency throughout the O–H stretching band of water.^47^ Even stronger an increase can be expected here considering the large frequency shift of the positive transient band with respect to the O–H band of pure **HFP**. As a consequence, the band area of the bleach could be significantly smaller than that of the positive band, and thus difficult to detect.

The early transient spectrum transforms in 4.2 ps into a spectrum, which is overall less intense and is characterized by a more downshifted positive band (green in Fig. 1B, EADS B in Fig. S5A†). This 4.2 ps time constant coincides well with the 3.3 ps time constant associated with the splitting of the C≡N bands of **ADA** in the S_1_ state and ascribed to an amplification of the symmetry-breaking *via* the strengthening of the H-bond at one cyano end and the concurrent weakening of the H-bond at the other cyano end.^29^ In the O–H stretching region, this should appear as a broadening of the positive transient band on both the low-frequency side (H-bond strengthening) and the high-frequency side (H-bond weakening). Taking the above-mentioned non-Condon effect into account, the broadening of the low-frequency side should be substantially more marked, in agreement with the measured spectrum.

The 110 ps time constant associated with the build-up of the late transient spectrum (blue in Fig. 1B, EADS C in Fig. S5A†) is identical to the excited-state lifetime of **ADA** in **HFP**.^29^

Therefore, this late spectrum, which consists of a negative feature around 3400 cm^–1^ and a weaker positive band at ∼3600 cm^–1^, should be due to vibrational modes of the solvent around **ADA** in the electronic ground state. Similar spectra are frequently observed at late times in IR-pump/IR-probe and 2D-IR experiments with H-bonded systems and are usually attributed to O–H vibration of hot molecules, *i.e.* of molecules with excited low-frequency modes.^48–51^ This characteristic shape is due to the ‘heating’ of the H-bonded solvent network, which causes the disruption and weakening of the H-bonds. This effect is responsible for the bleach at ∼3400 cm^–1^. The ‘hot’ O–H vibrators connected by weaker hydrogen bonds absorb at higher frequencies and have lower transition dipole moments due to vibrational non-Condon effects.^46–48^ This results in the weaker positive band around 3600 cm^–1^. The fact that a bleach is clearly visible at late time only is easily accounted for by considering that only those few **HFP** molecules H-bonded to **ADA** contribute to the earliest spectra, whereas the later spectra are due to the much larger number of **HFP** molecules surrounding **ADA** that absorb the energy, about 2.7 eV, released upon non-radiative decay of the S_1_ state.

**Fig. 3:**
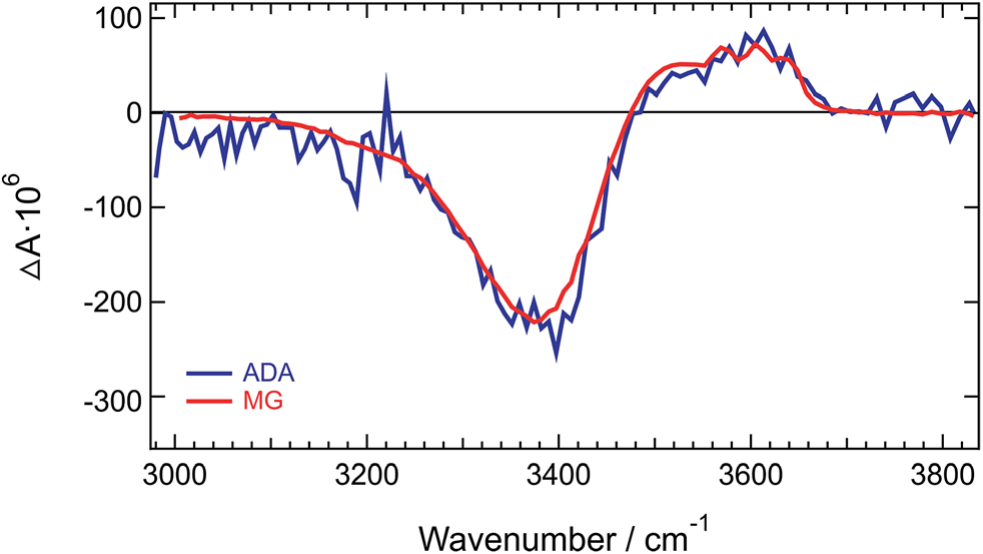
Normalized TRIR spectra measured at long time delays with **ADA** and malachite green (MG) in **HFP**.

Similar difference absorption spectra were obtained by subtracting the stationary IR spectrum of **HFP** at 20° C from those measured at higher temperatures (Fig. S7†). Moreover, TRIR spectra measured a few picoseconds after excitation of malachite green instead of **ADA**, are identical to the late spectra of **ADA** (Fig. 3 and S8†). This dye was chosen for the well-known ultrafast non-radiative decay of its S_1_ state through large amplitude motion of the phenyl substituents.^52–56^

The temperature elevation of the solvent involves two consecutive steps:^57–59^ (1) heat production, namely the non-radiative decay of the excited state and (2) the transfer of vibrational energy from the solute in the hot electronic ground state to the solvent, the so-called vibrational cooling. The later step depends on both solute and solvent and takes place on a timescale ranging from a few to a few tens of ps.^60–65^ In the case of **ADA**, vibrational cooling is faster than the 110 ps excited-state decay, which is the rate-determining step in the heat transfer to the solvent. After this temperature jump, the solvent keeps the same temperature until heat diffuses away from the irradiated spot on a microsecond timescale.^66^

### **ADA** in **HFP-d_2_** and **HFB**

**Fig. 4:**
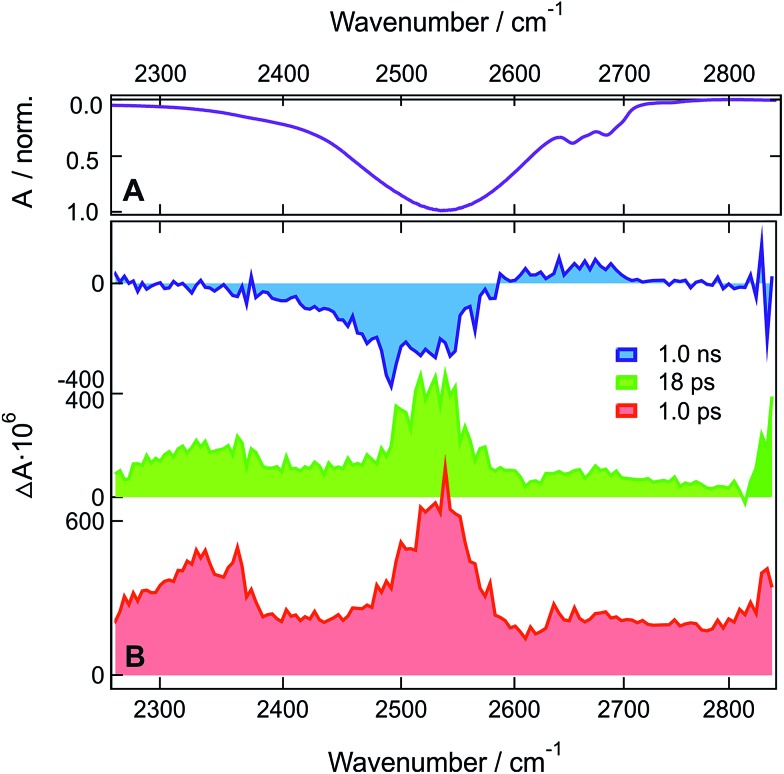
(A) Inverted stationary IR absorption spectrum of **HFP-d_2_** in the O-D stretching region. (B) Transient IR absorption spectra recorded at three representative time delays after 400 nm excitation of **ADA** in **HFP-d_2_**.

TRIR measurements with **ADA** in **HFP-d_2_** and **HFB** were performed in the C≡N stretching region (Fig. S10–S12†) as well as in the O-D (Fig. 4) and O–H (Fig. S13†) stretching regions. The spectral dynamics in the C≡N stretching region are similar to those observed in **HFP** and discussed in detail previously (see also ESI†).^29^ The only major difference is the longer excited-state lifetime of 660 ps in **HFB** compared to 130 and 110 ps in **HFP-d_2_** and **HFP**, respectively.

In **HFP-d_2_**, the early spectra in the O-D stretching region are dominated by two positive bands at ∼2350 and ∼2530 cm^–1^ (Fig. 4). Compared to the stationary absorption spectra of **HFP-d_2_**, these bands could be ascribed to solvents molecules that are strongly and weakly H-bonded to **HFP-d_2_**, respectively. Alternatively, these two distinct bands could also arise from the overlap of a broad positive band with a negative bleach centred around 2400 cm^–1^. Such a broad band could reflect the large distribution of H-bond strengths between the solvent molecules and the two cyano groups of **ADA** in the S_1_ state. The bleach, frequency down-shifted with respect to the stationary absorption spectrum of **HFP-d_2_**, could indicate that, before excitation of **ADA**, the solvent molecules responsible for the positive transient band were originally absorbing on the red side of the O–D stretch band, and were thus experiencing stronger-than-average H-bond interactions.

Contrary to **HFP**, the solvent molecules undergoing a weakening of the H-bond upon excitation of **ADA** contribute significantly to the transient spectrum in **HFP-d_2_**. This could indicate that non-Condon effects are less pronounced for the O–D than the O–H stretching mode, in agreement with previous studies in water.^46,67,68^ The positive bands undergo a partial decrease in ∼5 ps without significant changes in shape and then transform on a 100 ps timescale into the hot solvent spectrum. This latter step coincides well with the decay of the S_1_ state of **ADA** in this solvent. The TRIR spectra measured with **ADA** in **HFB** in the O–H stretching region are relatively similar to those in **HFP** and are discussed in the ESI.† The main difference is the slower excited-state decay, with a 660 ps time constant *vs.* 110 ps in **HFP**.

### Polarization-resolved measurements in **HFP**

**Fig. 5:**
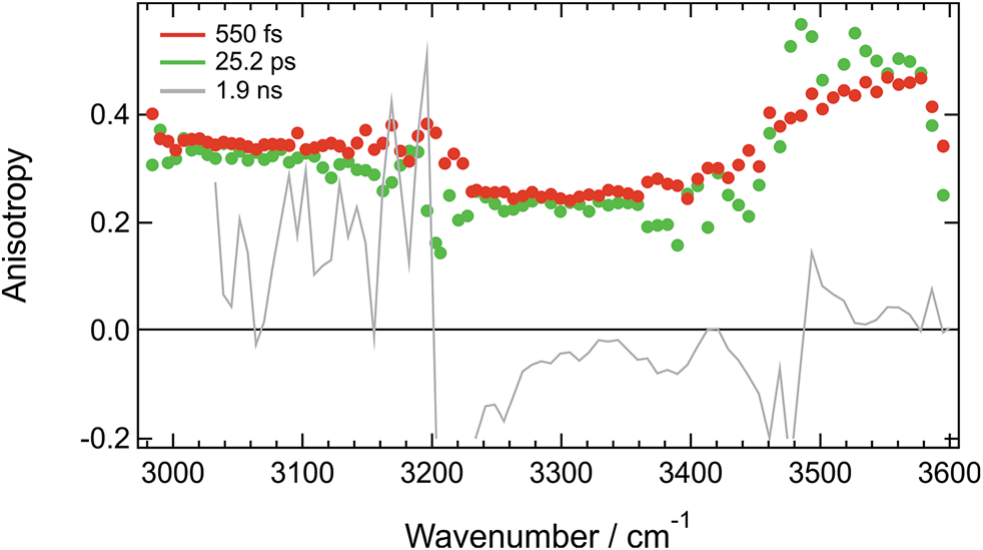
Spectral dependence of the anisotropy measured at three different time delays after 400 nm excitation of **ADA** in **HFP**.

Additional structural information on the tight H-bond complex between **ADA** and the solvent were obtained using polarization-resolved TRIR in **HFP** in the 3000–3600 cm^–1^ region. The anisotropy measured at three different time delays is shown in Fig. 5. Given the intrinsic weakness of the transient signal, about 5 × 10^5^ spectra had to be recorded at each pump-probe polarizations to extract reliable anisotropy values. Therefore, these measurements were only performed at few time delays.

Fig. 5 reveals that the early anisotropy between 3000 and 3200 cm^–1^, where the positive transient band is the most intense, amounts to 0.34 ± 0.02, whereas it is lower, *i.e.* 0.25 ± 0.02, between 3200 and 3500 cm^–1^ and is around 0.4 between 3500 and 3600 cm^–1^. The initial polarization anisotropy value of a TRIR band depends on the angle between the electronic transition dipole moment involved in the excitation and the probed vibrational transition dipole moment.^69–71^ Unless two transient bands with opposite signs contribute to the signal, the anisotropy for one-photon excitation ranges from –0.2 to 0.4, for perpendicular or parallel transition dipoles, respectively.^72,73^ As discussed above, the transient signal above 3500 cm^–1^ arises mostly from a S_2_ ← S_1_ electronic transition of **ADA**. Considering the symmetric and quadrupolar nature of **ADA**, both the S_1_ ← S_0_ and S_2_ ← S_1_ transition dipoles are predicted to be parallel and aligned along the main molecular axis, in full agreement with the initial anisotropy value of 0.4 found here. The 0.34 anisotropy in the 3000–3200 cm^–1^ region indicates that the hydroxyl groups of the **HFP** molecules bound to **ADA** are almost aligned along the molecular axis, as expected for H-bond interaction at a cyano group. The departure from 0.4 could be interpreted as a distribution of N···H–O angles. According to the wobbling-in-a-cone model,^74^ the half-width of this distribution would amount to about 19°. The lower anisotropy between 3200 and 3500 cm^–1^ is most probably due to the overlap of the induced absorption band with the bleach. According to Fig. 5, the anisotropy decreases by less than 10% during the first 25 ps. Assuming exponential decay, this decrease corresponds to anisotropy lifetime larger than 200 ps. Such long lifetime clearly points to a tight H-bond complex and not to a loose complex with rapid bond breaking and bond formation, which should lead to much faster anisotropy decay, similar to those found in hydrogen-bonded networks such as water or alcohols.^48,49,75,76^ Such slow anisotropy decay corresponds rather to the reorientational motion of the whole complex. A reorientational time of 540 ps was measured for **ADA** in isopropanol, which has a similar viscosity as **HFP** but where HBIND is not operative (Fig. S14†).

Despite the predicted anisotropy lifetime of more than 200 ps in **HFP**, no significant anisotropy could be recorded at 200 ps after excitation, when the TRIR spectrum is due to the hot solvent molecules. This indicates that the intrinsic anisotropy of the hot solvent spectrum is essentially zero. This is fully expected as this spectrum arises from a large number of solvent molecules with many different orientations of the O–H bond relative to the long axis of **ADA**.

### **ADA** in **HFP**/CHCl_3_ mixtures

The above-described results evidence the strong coupling between **ADA** in the S_1_ state and these superprotic solvents, in total agreement with a tight H-bonded complex. The shortening of the excited-state lifetime of **ADA** in this solvents reveals that the HBIND process is closely connected with the formation of this complex. However, these results do not allow determining whether the formation of the H-bond complex is a sufficient condition for HBIND to be operative. To find this out, measurements were performed with **ADA** in an aprotic solvent with different concentrations of **HFP**. CHCl_3_ was chosen as inert solvent because it is polar, aprotic and a very weak H-bond acceptor and donor.

**Fig. 6:**
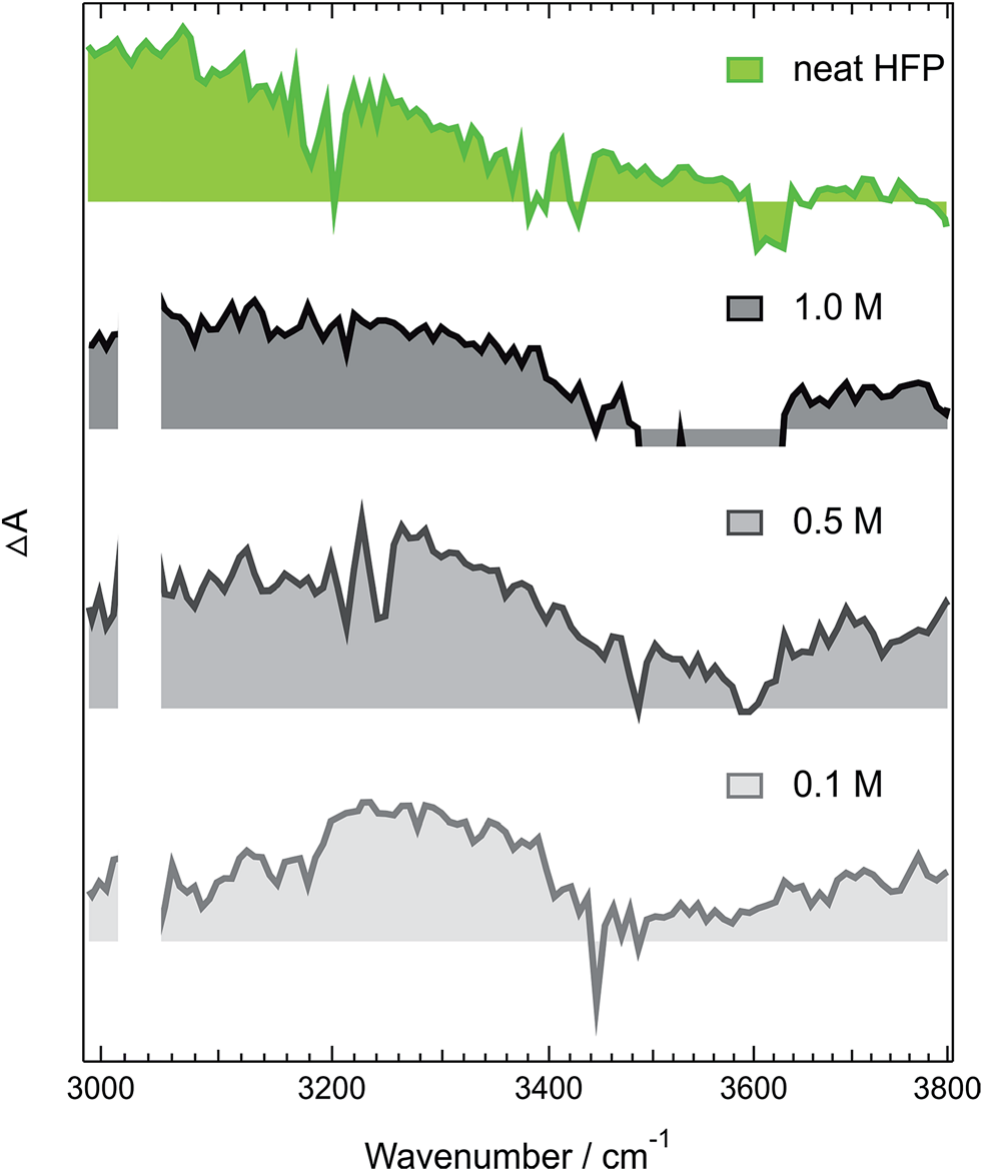
Intensity-normalized transient IR spectra recorded 1.7 ns after 400 nm excitation of **ADA** in CHCl_3_ and various concentrations of **HFP** (gray) compared with that measured 25 ps after excitation of **ADA** in pure **HFP** (green).

Like those in pure **HFP**, the TRIR spectra measured in the O–H stretching region with different concentrations of **HFP** in CHCl_3_ (0.1, 0.5 and 1.0 M) show the presence of the positive O–H stretching band of **HFP** at all three concentrations, pointing to the formation of an **ADA**/**HFP** complex (Fig. S18†). Such complex formation is also supported by the TRIR spectra in the C≡N stretching region as discussed in the ESI (Fig. S15–S17†). Contrary to the spectra measured in pure **HFP** (Fig. 1), the O–H band is still visible up to 2 ns (Fig. 6), pointing to a much longer excited-state lifetime of the complex, of the order of 1.5 ns according to global analysis (Fig. S19†). A consequence of this much longer lifetime than in pure **HFP** (110 ps) is that only a small fraction of the S_1_ energy is released as heat and dissipated into the solvent on a much slower timescale. Consequently, the hot solvent spectrum that dominates the late spectra in pure **HFP** is not visible here.

Fig. 6 reveals that the maximum of the O–H band shifts to lower frequencies with increasing **HFP** concentration, the lowest frequency being that measured in pure **HFP**. This effect could have several origins: (i) a higher probability to form a tight H-bond complex; (ii) H-bond interactions between the **HFP** molecule bound to **ADA** and nearby **HFP** molecules; and (iii) vibrational solvatochromism due to the higher dielectric constant of **HFP** compared to CHCl_3_.^77^ The latter phenomenon should not be dominant as this frequency downshift of the O–H band coincides with an increased splitting of the C≡N bands (see Fig. S10†). Vibrational solvatochromism would shift both C≡N bands toward the same direction.^43,45,78,79^

**Fig. 7:**
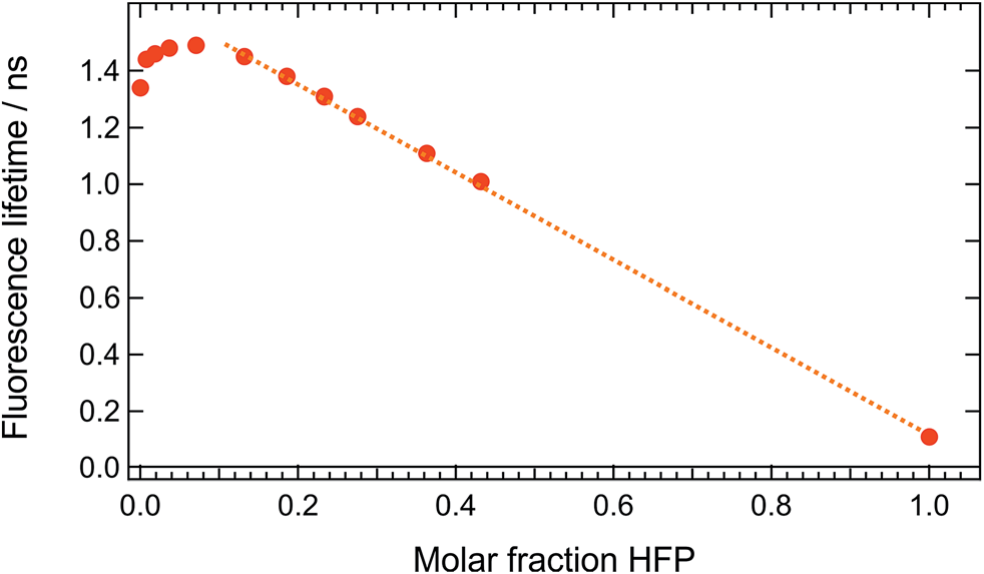
Dependence of the fluorescence lifetime of **ADA** on the mole fraction of **HFP** in CHCl_3_.

These measurements indicate that, up to 1 M **HFP** in CHCl_3_, the decay of the S_1_ state of **ADA** takes place on the ns timescale. The effect of **HFP** concentration on the fluorescence lifetime of **ADA** in CHCl_3_ was measured by time-correlated single-photon counting over a wide range of concentrations. At all mole fractions investigated, the fluorescence decay could be well reproduced with a single exponential function. Fig. 7 shows that the fluorescence lifetime increases from 1.35 ns in pure CHCl_3_ to 1.50 ns upon addition of 1 M **HFP** corresponding to a mole fraction of **HFP**, *x*_HFP_, of 0.08. At higher mole fractions, the fluorescence lifetime decreases linearly with *x*_HFP_ down to 110 ps in pure **HFP**.

**Fig. 8:**
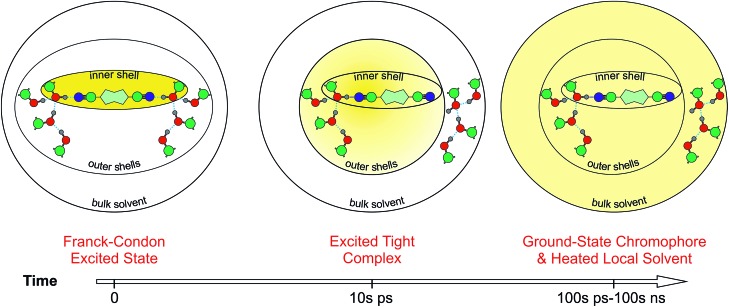
Schematic picture of the HBIND process. The yellow shading represents the excitation energy, initially localized on the chromophore and finally dissipated into the environment.

This figure reveals unambiguously that the HBIND process is not operative at low **HFP** concentration (≤1 M). The fluorescence lifetime of **ADA** was found to increase from 1.0 ns in the apolar cyclohexane to 1.5 ns in the highly polar acetonitrile (Fig. S2†).^29^ The increase of the fluorescence lifetime with **HFP** concentration measured here below 1 M **HFP** can be explained by the increase of polarity of the solvent mixture around **ADA**. On the other hand, the decrease of the fluorescence lifetime at *x*_HFP_ > 0.1 can be assigned to the increased efficiency of the HBIND process.

## Discussion

A rather precise picture of the HBIND mechanism can be deduced from the above results. The HBIND process was previously observed with dyes containing an H-bond accepting group and characterized by an S_1_ state with a substantial charge-transfer character.^13–22,24^ As the electronic density on the H-bonding site of the dye was increasing upon charge-transfer excitation, HBIND was proposed to be associated with a strengthening of the H-bonding interaction with the solvent.^21,24^

Unambiguous experimental confirmation of such H-bond strengthening was obtained here by directly probing the relevant solvent modes. The presence of the positive O–H stretching band of the solvent in the early TRIR spectra evidences the prompt strengthening of the H-bond between **ADA** and the solvent. If the strength of these H-bond interactions remained unchanged upon S_1_ ← S_0_ excitation, no transient O–H band would be observed. As shown previously, dipolar solvation leads to an asymmetric distribution of the excitation over **ADA**, that leads to a different basicity of the two cyano groups.^29^ The first stage of this symmetry breaking is due to the inertial motion of the solvent and takes place within 100-200 fs. Thus photoexcitation of **ADA** leads to a quasi-instantaneous change of H-bond strength with the solvent. H-bonding should become stronger on one CN end of **ADA** and weaker at the other. This distribution of H-bond strengths explains the broad width of the transient O–H band. The TRIR spectra show that the O–H band shifts toward lower frequency on a few ps timescale. This is a direct consequence of a further strengthening of the H-bond with the most basic CN group of **ADA**. The formation of the tight H-bond complex also manifests in the C≡N stretching region by the presence of the two strongly split bands of **ADA** in the S_1_ state (Fig. S10†).

The frequency splitting of these two bands, Δ*ν̄*_C≡N_ (S_1_), measured in all superprotic solvents is listed in Table 1. For comparison this value in methanol, where only a loose H-bond complex is formed is also given. Additionally, the C≡N stretching frequency of **ADA** in the ground state, *ν̄*_C≡N_ (S_0_), determined from the position of the ground-state bleach in the TRIR spectra is listed. This table indicates that both Δ*ν̄*_C≡N_ (S_1_) and *ν̄*_C≡N_ (S_0_) are well correlated with the Kamlet–Taft parameter *α*, pointing to an increase with *α* of the strength of the H-bond interaction between the solvent and **ADA** in both the S_0_ and the S_1_ states. However, Table 1 reveals that the excited-state lifetime of **ADA**, *τ*_S_1__, does not correlate well with *α*. One can thus conclude that, although strong H-bond solute–solvent interactions are required for HBIND to be operative, they are not sufficient to make this process efficient. If this were the case, the excited-state lifetime of **ADA** in **NFB** (*α* > 2) should be shorter than in **HFP** (*α* = 1.96), contrary to the observation. This conclusion is also supported by the measurements in CHCl_3_/**HFP** mixtures, which reveal that, although a tight H-bonded **ADA**–**HFP** complex is formed in CHCl_3_, the HBIND process is not operative at **HFP** concentrations below 1 M.

**Table 1:** Solvent dependence of the splitting of the excited-state C≡N bands, Δ*ν̄*_C≡N_ (S_1_), of the C≡N stretching frequency in the ground state, *ν̄*_C≡N_ (S_0_), and of the excited-state lifetime of ADA

Solvent	*α* ^*a*^	*ρ*_OH_ ^*b*^/nm^–3^	Δ*ν*_C≡N_ (S_1_)^*c*^/cm^–1^	*ν*_C≡N_ (S_0_)/cm^–1^	*τ*_S_1__/ps
Methanol	0.98	14.9	∼50	∼2225	1600
Chloroethanol	1.28	8.4	∼70	2227	1400
Trifluoroethanol (**TFE**)	1.51	8.4	104	2235	290
Hexafluoro-*tert*-butanol (**HFB**)	<1.96	4.9	104	2239	660
Hexafluoroisopropanol (**HFP**)	1.96	5.7	131	2242	110
Hexafluoroisopropanol-d_2_ (**HFP-d_2_**)	>1.96	5.7	137	2245	130
Nonafluoro-*tert*-butanol (**NFB**)	>2	4.3	115	2247	950

^*a*^Kamlet–Taft parameter, from ref. 26.

^*b*^Density of OH groups calculated as the inverse of the volume occupied by a single solvent molecule, using the molecular weight and the density of the solvent.

^*c*^The values in methanol, chloroethanol, **TFE** and **NFB** are taken from ref. 29.

Consequently, the shortening of the fluorescence lifetime is due to a cooperative effect of the protic solvent molecules and involves the formation of a H-bond network. In such case, the excited dye is coupled to a large H-bond network and not only to a single solvent molecule. Therefore, the density of states of **ADA** + H-bond network is significantly larger than that of **ADA** alone, and the S_1_ → S_0_ internal conversion is strongly accelerated.

This importance of the H-bond network for the HBIND process can explain the relatively long excited-state lifetime of 950 ps measured in **NFB**, despite its *α* parameter larger than that of **HFP**,^80^ and the unambiguous formation of a tight H-bonded complex.^29^ A dense H-bond network requires the solvent molecules to act as both H-bond donor and acceptor. With **NFB**, this is only possible if the molecules organize in small clusters, consisting of a core, where the OH groups are close enough to interact, surrounded by the perfluoroalkyl groups. Therefore, because of its bulkiness, **NFB** cannot organize in an extended network. More generally, the density of the OH groups, *ρ*_OH_, which can be considered as a measure of the ability to form an H-bond network, decreases when going to alcohols with long alkyl or branched chains.^81^ In conventional non-halogenated alcohols, the *α* parameter follows the same trend as *ρ*_OH_ and decreases continuously when going from water to propanol. This reduced ability of these bulky solvents to form a dense H-bond network is also visible in the stationary IR absorption spectrum in the O–H stretching region, that point to the presence of free O–H groups (Fig. 1, 4 and S9†).

This parallel variation of *α* and *ρ*_OH_ can explain the clear dependence of the non-radiative rate constant on *α* observed in previous studies, which were only performed in water (*α* = 1.17) and in non-halogenated alcohols (*α* < 1).^18,21,23,24^ For this reason, *α* was considered to be an adequate and sufficient parameter for predicting the efficiency of the HBIND process. However, it should be noted that in ref. 24, the fluorescence lifetime of the dye was found to decrease continuously when going to alcohols longer than propanol, up to hexanol, although *α* remains almost constant in these solvents, around 0.8. This decrease of the fluorescence lifetime was ascribed to the effect of solvent polarity. However, the decrease of *ρ*_OH_ should be envisaged as an alternative explanation.

The H-bond interactions with **ADA** in the S_1_ state are weaker than with the previously investigated dyes because the H-bond accepting groups are nitrile and not carbonyl or nitro groups. Therefore, HBIND is only operative in superprotic solvents with *α* > ∼1.3, *i.e.* in halogenated alcohols. For these solvents, however, *ρ*_OH_ decreases with increasing *α*, because the solvent molecules become bulkier. Therefore, although the H-bond interactions between one solvent molecule and the excited dye strengthen as *α* increases, the HBIND efficiency decreases, because of the diminishing ability of the solvent to form a H-bonded network. As a consequence, the reduction of the excited-state lifetime of **ADA** is determined by the ability of the cyano group to couple not to a single molecule but to a large number of H-bonded solvent molecules. **HFP** provides the best coupling out of the fluorinated alcohols investigated here and therefore the lifetime is the shortest in this solvent (110 ps). This corresponds to a shortening by more than one order of magnitude compared to isopropanol. For **TFE**, the weaker H-bonding to **ADA** is compensated by a larger the H-bond network. Consequently, the lifetime shortening is still substantial (300 ps, *i.e.* 5 times shorter than in ethanol). Finally, **NFB** makes the strongest H-bond but has very limited ability to organise in a large network. Consequently, the lifetime is less affected in this solvent (950 ps, 30–40% less than in *tert*-butanol). This illustrates the importance of the solvent network, quantified by *ρ*_OH_, additionally to that of the Kamlet–Taft parameter *α.* More subtle effects, like those associated with the structure of the solvation shell,^82^ could also play a role here. However, their evidence would require molecular dynamics simulations of **ADA** in the various solvents, and would go far beyond the main scope of the present investigation.

Beside *α* and *ρ*_OH_, the prompt change of H-bond strength upon photoexcitation is an important parameter controlling the HBIND efficiency. The larger this change, the further from equilibrium is the H-bond directly after excitation of the solute. Such large displacement of the S_1_ and S_0_ potentials along the H-bond coordinate favours large Franck–Condon factors for internal conversion. Internal conversion will be all the more efficient if the solvent forms a dense H-bond network. Both strengthening and weakening of the H-bond upon excitation lead to displaced potentials, but only bond strengthening warrants sufficient coupling of the excited dye to the H-bond network.

Fig. 8 presents a schematic picture of the HBIND process. Excitation energy is first localized on the chromophoric unit and the directly H-bonded solvent molecules that form the inner-shell. On the timescale of solvation, both the inner-shell and outer-shell solvent molecules equilibrate to form a tight H-bond complex coupled to the solvent H-bond network. Electronic energy is then converted into vibrational energy of the inner and outer-shell and finally dissipated into the bulk solvent.

## Conclusions

Solute-pump/solvent-probe TRIR spectroscopy was applied here to investigate, from the solvent side, H-bonding interactions between an excited chromophore and the solvent. This approach allowed direct visualization of the changes of H-bond strength that take place upon photoexcitation of the solute. This was shown here with the H-bond interactions between superprotic solvents and the cyano groups of **ADA**. By performing such measurements with **ADA** in pure solvents and in solvent mixtures, we could obtain a much deeper understanding of the HBIND mechanism. The latter can be viewed as an enhanced internal conversion of an excited complex consisting of the solute molecule connected to a H-bond solvent network. As a consequence, the number of energy accepting vibrational modes is dramatically increased relative to that of the naked solute.

For HBIND to be operative, the following conditions should be fulfilled for the solute: (i) it should possess H-bond accepting group(s), and (ii) the electronic density on this group should increase upon photoexcitation, *i.e.* the transition should have a substantial charge-transfer character. For the solvent, the HBIND efficiency depends on (i) its H-bond donating ability, quantified by the Kamlet–Taft parameter *α*, and (ii) its ability to form an extended H-bond network, quantified by the density of OH groups, *ρ*_OH_. In normal alcohols, these two properties increase approximately in parallel with decreasing the size of the alkyl group until water, where *ρ*_OH_ reaches its maximum value. Further increase of *α*, achieved by going to halogenated alcohols is accompanied by a decrease of *ρ*_OH_, and eventually by the decrease of the HBIND efficiency. In this respect, water seems to be one of the most efficient solvents for HBIND, given its unique ability to form an extended highly coupled H-bonded network.

Organic fluorophores are quite generally known to have a smaller fluorescence quantum yield in water than in organic solvents, this being a major problem in the quest for new fluorescent probes for biological applications. In some cases, this can be due to the occurrence of excited-state proton transfer or aggregation phenomena. However, in all the other cases, occurrence of the HBIND should be considered. On the other hand, this HBIND process could be advantageously exploited to develop specific fluorescent probes that only emit in non-aqueous environments.
