# Supplementary material for: Direct local solvent probing by transient infrared spectroscopy reveals the mechanism of hydrogen-bond induced nonradiative deactivation
Source: Chem Sci. 2017 May 16;8(7):5057–66. doi: 10.1039/c7sc00437k (PMC5613230; doi:10.1039/c7sc00437k)
Supplement: Supplementary file 1 [file SC-008-C7SC00437K-s001.pdf]

Electronic Supplementary Material (ESI) for Chemical Science.
This journal is © The Royal Society of Chemistry 2017

Supporting Information

Direct Local Solvent Probing by Transient Infrared Spectroscopy Reveals the Mechanism of Hydrogen-Bond Induced Nonradiative Deactivation

Bogdan Dereka and Eric Vauthey*

Department of Physical Chemistry, University of Geneva, 30 Quai Ernest-Ansermet, CH-1211 Geneva 4, Switzerland

Eric.Vauthey@unige.ch

1. Experimental
    1.1 Samples S2
    1.2 TRIR spectroscopy S2
2. Basic photophysics S4
3. Transient electronic absorption spectroscopy S6
4. Steady-state and transient infrared measurements in pure solvents
    4.1 HFP and acetone S7
    4.2 HFP-d_2 and HFB S10
    4.3 Polarization-resolved measurements in isopropanol S14
5. Transient infrared measurements in HFP/$CHCl_3$ mixtures S15
6. References S21

1. Experimental

1.1 Samples

2,6-di(4-cyanophenyl)-1,5-di(4-methylphenyl)-3,4-dihydropyrrolo [3,2-*b*]pyrrole, **ADA**, was provided by Prof. D. Gryko from the Institute of Organic Chemistry of the Polish Academy of Sciences in Warsaw and synthesized as described in ref S1. Malachite green (MG) was used as an oxalate salt and was used as received (Radiant Dyes). Chloroform, hexafluoroisopropanol (HFP), hexafluoroisopropanol-d2 (HFP-d_2) and hexafluoro-tert-butanol (HFB) were purchased from Sigma-Aldrich or Acros Organics, were of 99.0 % or higher purity, and were used as received. Highly fluorinated solvents contain large amounts of dissolved oxygen. To avoid oxygen quenching and formation of long-lived radical species, the solvents were thoroughly purged with nitrogen gas before addition of **ADA**.

The steady-state infrared spectra of pure solvents were obtained using both FTIR and TRIR instruments (see below) and were found to be identical. The steady-state IR spectra of these solvents were not affected by the presence of **ADA** at concentrations within the 100-300 μM range.

1.2 TRIR spectroscopy

Femtosecond TRIR spectra were obtained with the setup described in detail in reference S2 and based on a Ti:Sapphire amplified system (Spectra Physics, Solstice) producing 100 fs pulses at 800 nm and 1 kHz. Excitation was performed with 0.2-0.4 μJ pulses at 400 nm produced by frequency doubling a fraction of the amplifier output. The linearity of the signal amplitude with respect to excitation energy was checked before each experiment, and proper adjustment of the pump intensity was made to ensure the maximum signal within the linear response regime. The polarization of the pump beam was controlled with a Glan-Taylor polarizer and a zero-order half-wave plate. The pulses were focused on the sample to a 350 μm spot resulting in an irradiance of 0.05-0.10 mJ/cm^2. Mid-IR probe pulses in the 2.6-4.8 μm range were generated by difference frequency mixing of the output of an optical parametric amplifier (Light Conversion, TOPAS-C with NDFG module) pumped at 800 nm. The polarization of the IR beam was controlled using a wire-grid polarizer. Two horizontally polarized IR beams were produced upon reflection on a CaF_2 wedge and focused onto the sample to a 140 μm diameter spot. One of the

beams was overlapped with the pump beam, whereas the second one was used as a reference. Both IR beams were focused onto the entrance slit of an imaging spectrograph (Horiba, Triax 190, 150 lines/mm) equipped with a liquid nitrogen cooled 2 x 64 element MCT array (Infrared Systems Development), giving a resolution of 3-4 cm^{-1} (C≡N, O-D) and 7-10 cm^{-1} (O-H). The instrument response function, as determined from the cross-correlation of the pump and probe beams in a silicon wafer, was about 300 fs.

The sample area and the detection system were placed in a box that was purged with water-free and carbon dioxide-free air for at least one hour before and during each experiment. The average of 2000-3000 signal shots was taken to collect one data point with the polarization of the pump at the magic angle to that of the IR pulse or with parallel and perpendicular polarizations for anisotropy measurements. This procedure was carried out for at least twenty times. To provide a new sample solution for each shot, a flow cell as described in reference S3 was used. The key feature enabling direct solvent probing was to use very thin spacers, namely 12 and 25 μm thick teflon spacers. The sample absorbance at 400 nm was about 0.03-0.05 over the optical pathlength. Significantly higher absorbance could be achieved with malachite green due to its better solubility. No significant sample degradation was observed throughout the experiment. To avoid changes due to the evaporation of chloroform in the experiments with $CHCl_3$/HFP mixtures, sufficiently large sample reservoirs (10 mL) were used.

Given the bandwidth of the IR pulses (400 nm), transient spectra recorded in three to eleven, ca. 100-150 cm^{-1} wide, spectral windows were merged to cover the 2080-3840 cm^{-1} region (the exact region depending on the solvent). All data sets were first compared to detect possible problems during the measurements. The spectra were then averaged and the different windows merged to obtain a single spectrum. The resulting spectra were then compared, and regions without signal were inspected to identify the baseline. No tail matching had to be carried out.

2. Basic photophysics

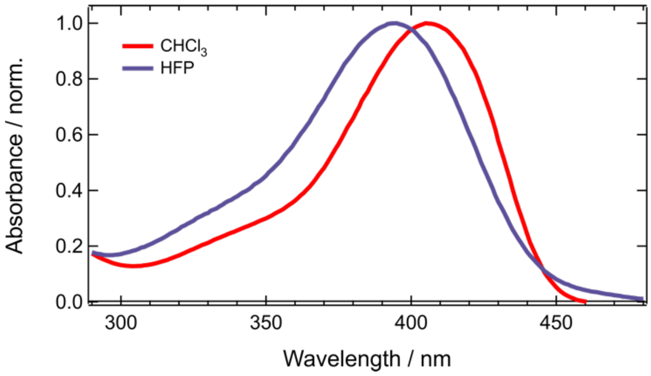

Figure S1. Steady-state electronic absorption spectra of **ADA** in HFP and chloroform. The difference in the band is due to the difference of refractive indices of these two solvents (n_D(HFP) = 1.275, n_D($CHCl_3$) = 1.350) and, thus, to different magnitudes of the dispersion interactions.

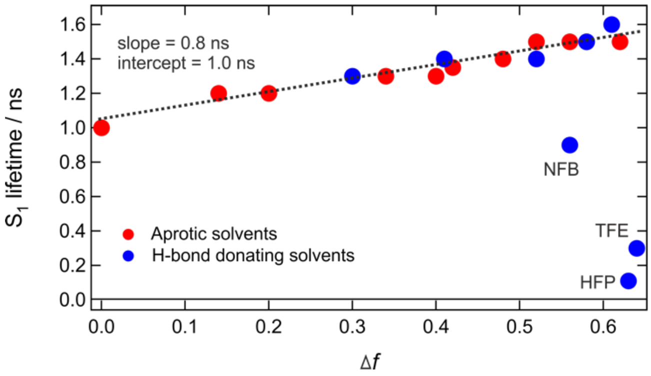

Figure S2. Dependence of the S_1 state lifetime of **ADA** on the Onsager polarity function $\Delta f = f(\varepsilon) - f(n^2)$ in 20 solvents. Tables with the lifetimes can be found in ref.S4. Clear linear dependence is observed for all the solvents except the superprotic ones. The lifetime depends only on the solvent polarity, increasing with the latter (from 1.0 ns in cyclohexane to 1.5 ns in acetonitrile). This effect is due to increasing energy gap between the S_1 and T_n excited states that impedes intersystem crossing as discussed in ref.S4. Only 18 points are seen in the graph because of the overlap of a few points. The dashed line represents a linear fit to the data performed without taking into account three ‘outliers’ for which the formation of tight H-bound complex is evident.

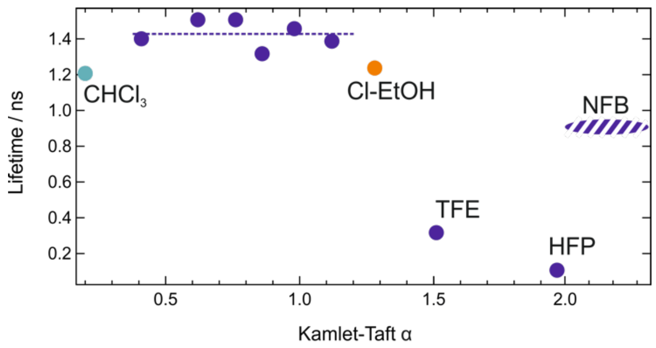

Figure S3. Dependence of the S_1 state lifetime of **ADA** on the Kamlet-Taft α parameter in protic solvents. There is no significant dependence on the H-bond donating ability of the solvent for $\alpha < 1.3$. When $\alpha > 1.3$ (starting from chloroethanol (Cl-EtOH)) the formation of tight H-bonded complex takes place and the lifetime is reduced. The lifetime in chloroform is smaller because of its lower polarity (see Figure S2).

Table S1. Radiative (k_r), non-radiative (k_{nr}) rate constants and fluorescence quantum yield (φ) of **ADA** in 'superprotic' solvents and in a 'standard' aprotic medium polar solvent for comparison.

**Solvent**	**φ, %**	**k_r, s^{-1}**	**k_{nr}, s^{-1}**
TFE	12	$4.1 \cdot 10^8$	$3.0 \cdot 10^9$
HFP	5	$4.5 \cdot 10^8$	$8.6 \cdot 10^9$
NFB	35	$4.0 \cdot 10^8$	$7.4 \cdot 10^8$
Aprotic medium polar	70-80	$5.7\text{-}5.8 \cdot 10^8$	$1.6\text{-}1.8 \cdot 10^8$

Table S1 lists the values of the radiative, k_r, and non-radiative rate constants, k_{nr}, extracted from the fluorescence quantum yield and fluorescence lifetime measurements in superprotic solvents and in a standard aprotic solvent of medium polarity. The k_r values are the same within 10%, whereas the k_{nr} values are drastically different in the 'superprotic' solvents and vary by a factor of 4. In aprotic solvents, k_r is larger by ~50% than in the 'superprotic' solvents. The small decrease of k_r upon increasing polarity of the aprotic solvents can be attributed to the decreasing S_1-S_0 gap.[S5] The substantially lower k_r value in 'superprotic' solvents points to a different nature of the emitting species, which is an asymmetric tight H-bonded complex. The non-radiative rate constant is higher in these alcohols by a factor of 5-20 compared to aprotic solvents. Surprisingly, the lowest non-radiative

rate constant is in NFB while the highest is in HFP. This non-radiative mechanism is clearly determined by hydrogen bonding although the correlation is not as simple as a mere Kamlet-Taft α could reveal (see the main text).

3. Transient electronic absorption spectroscopy

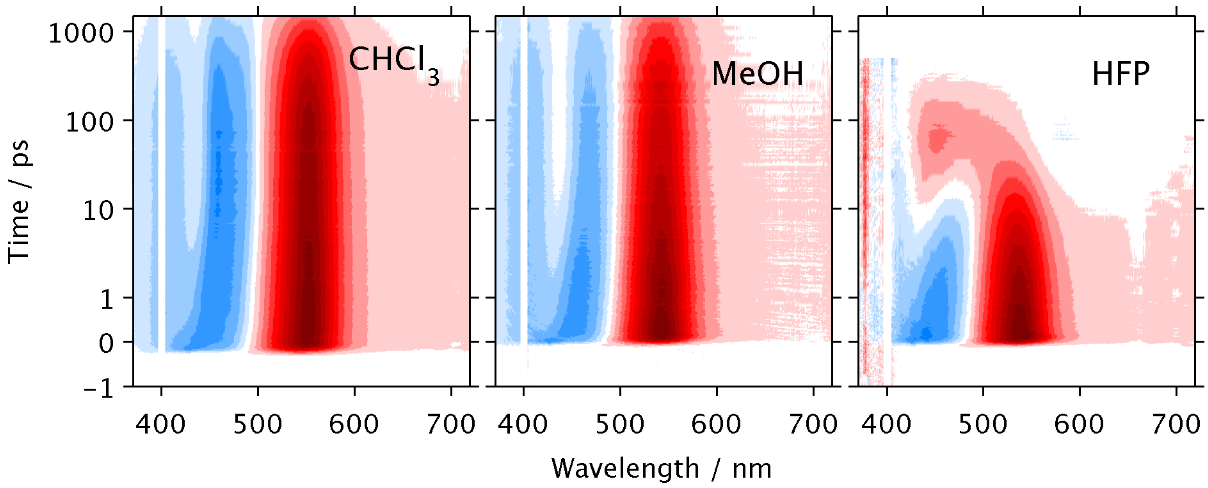

Figure S4. Temporal evolution of the transient electronic absorption measured upon 400 nm excitation of **ADA** in chloroform, methanol and HFP. Positive signals due to excited-state absorption (ESA) are in red and negative signals due to ground-state bleach (GSB) and stimulated emission (SE) are in blue. A clear upshift of the $S_n \leftarrow S_1$ ESA in the superprotic HFP is observed on a tens of picosecond timescale due to the stabilization of the S_1 state of **ADA** upon formation of a tight H-bonded complex.

4. Steady-state and transient infrared measurements in pure solvents

4.1 HFP and acetone

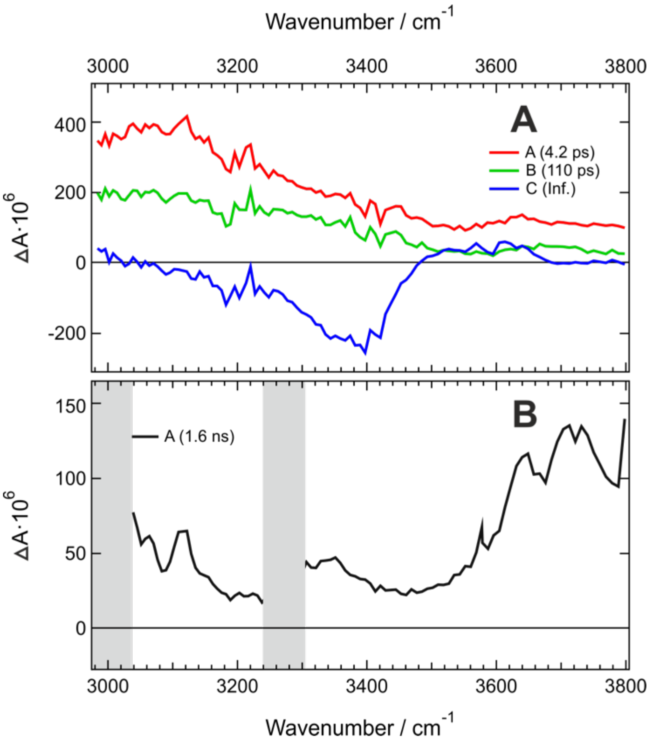

Figure S5. Evolution-associated difference spectra (EADS) obtained from global target analysis of the TRIR spectra recorded with **ADA** in HFP **(A)** assuming an A→B→C→S_0 scheme and in acetone **(B)** assuming an A→S_0 scheme.

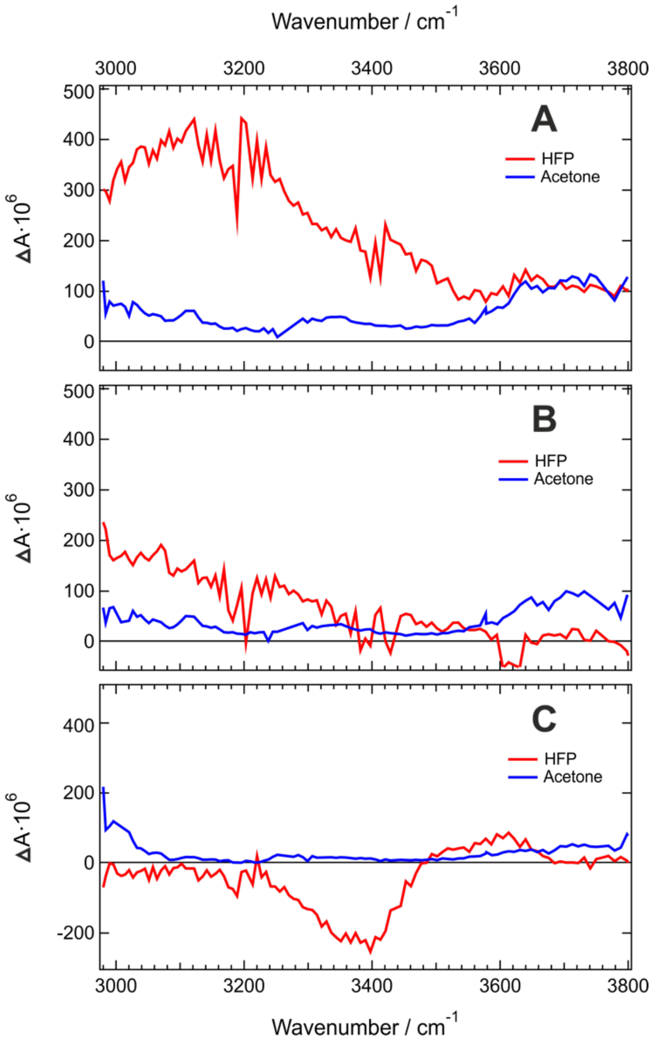

Figure S6. Comparison of the TRIR spectra in the O-H stretch region upon photoexcitation of **ADA** in HFP and acetone at early (**A**, 550 fs for HFP / 750 fs for acetone), intermediate (**B**, 25 ps for HFP / 20 ps for acetone) and late time delays (**C**, 1.9 ns for HFP / 1.6 ns for acetone). The spectra in both solvents were acquired under identical experimental conditions (concentration, optical pathlength and excitation intensity), and the intensities in the two solvents can be directly compared. Note the different y-axis in panel **C**.

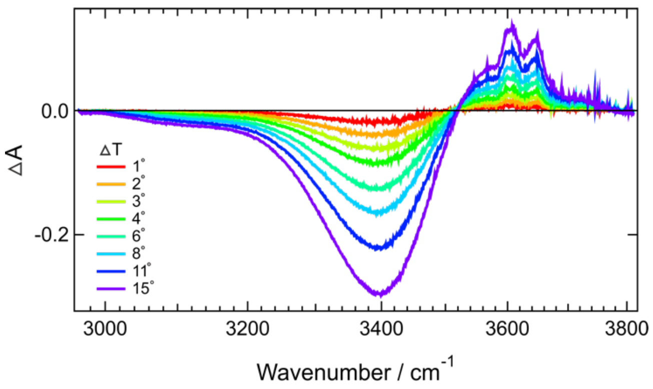

Figure S7. Thermal difference steady-state infrared spectra of pure HFP. The legend indicates the thermal difference associated with each spectrum.

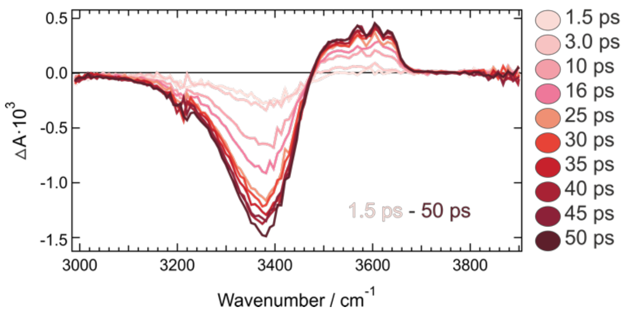

Figure S8. TRIR spectra in the O-H stretch region measured at different time delays after excitation of malachite green in HFP.

4.2 HFP-d_2 and HFB

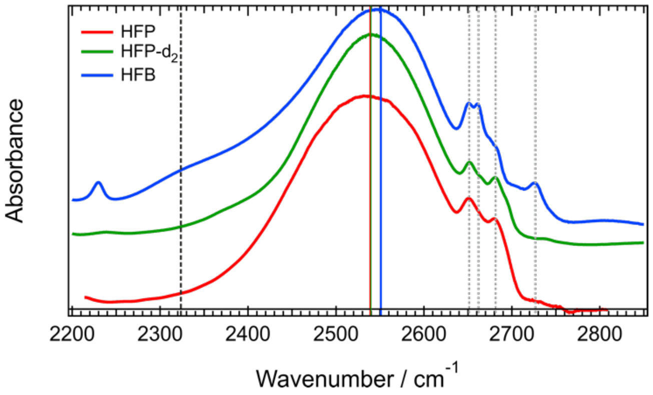

Figure S9. Steady-state IR absorption spectra of HFP, HFP-d_2 and HFB. The wavenumber axis corresponds to that of HFP-d_2 and was scaled by a factor of 0.738 for HFP and HFB in order to bring the three spectra on a common axis for direct comparison. This scaling factor corresponds to the ratio of O-D to O-H stretch frequencies in the gas phase. The spectra are vertically offset for clarity. The solid vertical lines indicate the position of the band maximum. The dotted grey lines show the positions of free OH groups corresponding to different conformers with respect to C-H groups. The dashed black line indicates a weak shoulder present in HFB and corresponding to the strongest H-bonded OH oscillators. Overall, the spectra are very similar and are identical in the free OH group region. The heterogeneity of the red-shifted H-bonded OH vibrators is more pronounced in HFB, as evidenced by the increased bandwidth and the additional shoulder indicated by the dashed black line. The band maximum of HFB is also 10 cm^{-1} upshifted compared to the other two solvents, pointing slightly weaker average H-bonding in this solvent.

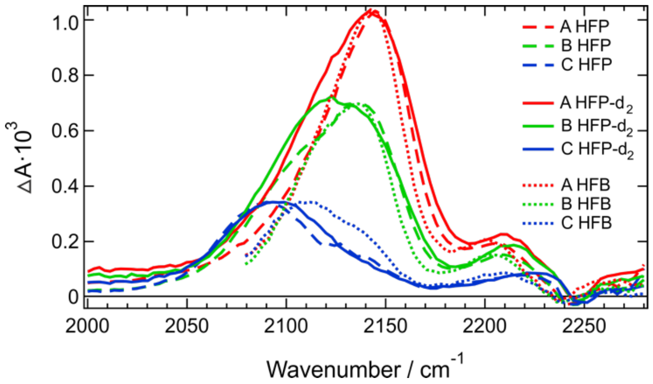

Figure S10. EADS obtained from the target global analysis of TRIR data in the C≡N stretching region measured with **ADA** in HFP, HFP-d_2 and HFB and assuming an A→B→C→S_0 scheme.

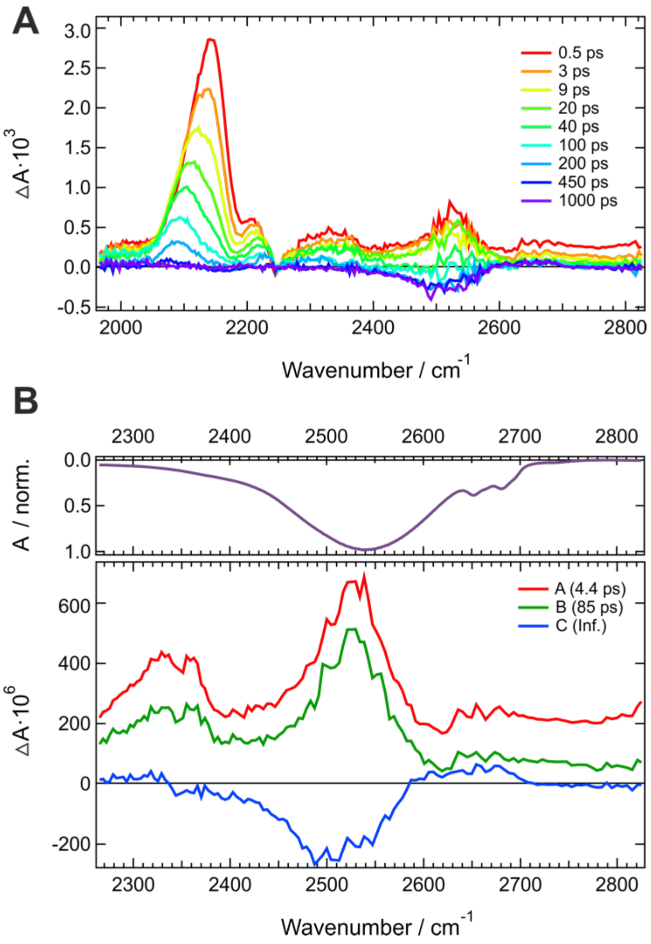

Figure S11. (A) TRIR spectra in the C≡N (below 2250 cm^{-1}) and O-D stretching regions measured with **ADA** in HFP-d_2. **(B)** Inverted steady-state infrared spectrum of HFP-d_2 and evolution-associated difference spectra (EADS) obtained from global target analysis of the TRIR spectra in the O-D stretching region recorded with **ADA** in HFP-d_2 assuming an A→B→C→S_0 scheme.

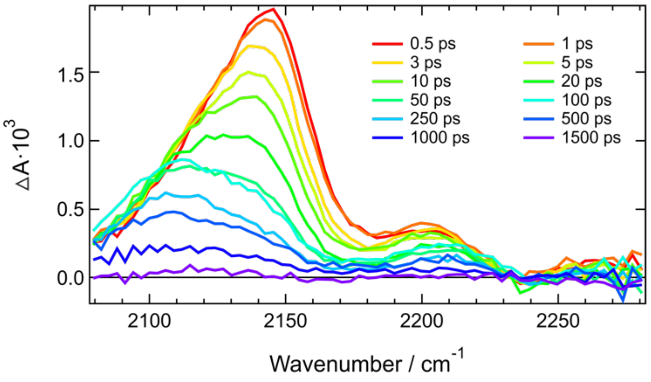

Figure S12. TRIR spectra in the C≡N stretch region measured at different time delays after excitation of **ADA** in HFB.

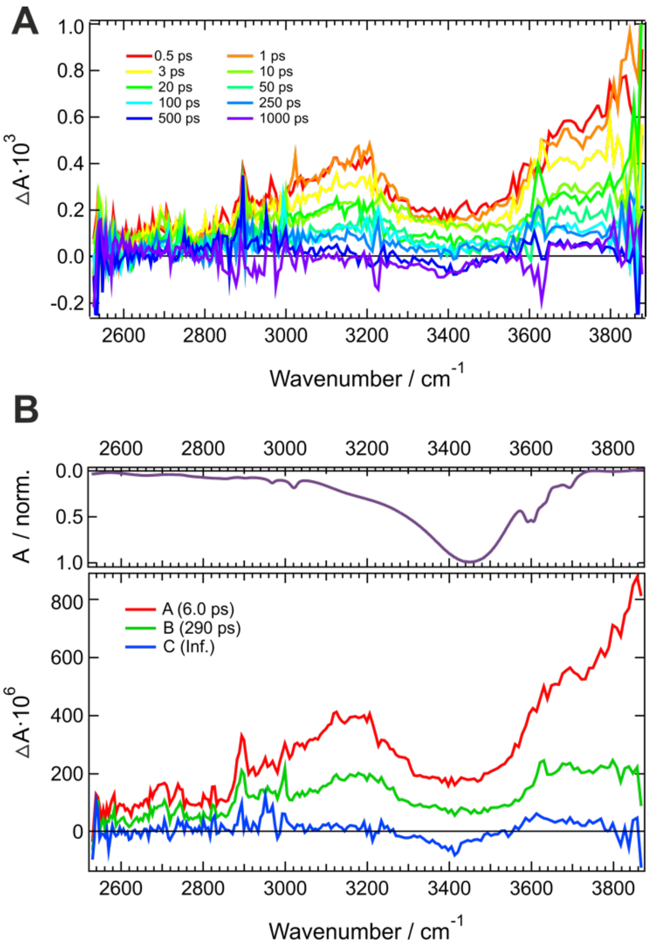

Figure S13. (A) TRIR spectra in an extended O-H stretching region measured with **ADA** in HFB. **(B)** Inverted steady-state infrared spectrum of HFB (top) and evolution-associated difference spectra (EADS) obtained from global target analysis of the TRIR spectra recorded with **ADA** in HFB (panel A) assuming an A→B→C→S_0 scheme (bottom).

Global target analysis of the TRIR data in HFP-d_2 and HFB recorded over the entire spectral window (i.e. C≡N + O-H (or O-D) stretching regions) required three consecutive steps, whereas only two steps where sufficient to reproduce the data in the O-D and O-H stretching region. The strongest spectral changes during the first ~50 ps take place in the C≡N stretching region (figures S10-S12). They are similar to those observed in HFP and discussed in detail previously.[S4] In brief, the presence of two C≡N bands is due to the ultrafast symmetry breaking of the S_1 state of **ADA**, with the most intense band, ESA1, associated with the cyano group bearing most of the excitation and the weak band, ESA2, due to the other cyano group. During the first few ps, the splitting of ESA1 and ESA2 increases continuously due to a further symmetry breaking brought about by diffusive solvent relaxation and the equilibration of the H-bonds between the cyano ends of **ADA** and the solvent. Afterward, these

bands decay on a ~20 ps timescale and are replaced by two new bands, ESA3 and ESA4, which are characterized by a larger splitting. These bands are associated with an asymmetric complex consisting of solvent molecule(s) tightly bound to the cyano group located on the branch of **ADA** bearing most of the S_1 excitation. Finally, the decay of these two bands on a longer timescale reflects the decay of the excited-state population to the ground state. Similar spectral dynamics to that observed in HFP in the C≡N stretch region take place in HFP-d_2 and HFB (figures S10-S12). The main difference is the longer excited-state lifetime of 660 ps measured in HFB, compared to 110 and 130 ps in HFP and HFP-d_2, respectively.

The TRIR spectra measured with **ADA** in HFB in the O-H stretching region show the $S_2 \leftarrow S_1$ absorption band of **ADA** above 3400 cm^{-1} as well as a positive band due to the solvent with a maximum around 3160 cm^{-1} and extending below 2800 cm^{-1} (Figure S13). Here again, this band can be ascribed to HFB molecules whose H-bond with **ADA** strengthens upon excitation. Because of the non-Condon effects, the presence of the $S_2 \leftarrow S_1$ band and possible overlap with a bleach at 3400 cm^{-1}, the contribution of the solvent molecule undergoing a weakening of the H-bond upon excitation of **ADA** is not visible like in HFP. The broad 3160 cm^{-1} band decays partially and broadens further on the low-frequency side during the few ps and then evolves to the hot solvent spectrum on a timescale similar to the decay of the S_1 state. Compared to HFP and HFP-d_2, the relative amplitude of the hot solvent spectrum is significantly smaller with HFB. This is in agreement with the longer S_1 state lifetime of **ADA** in this solvent. Consequently, the fluorescence quantum yield is higher and the amount of heat deposited into the solvent is correspondingly smaller.

4.3 Polarization-resolved measurements in isopropanol

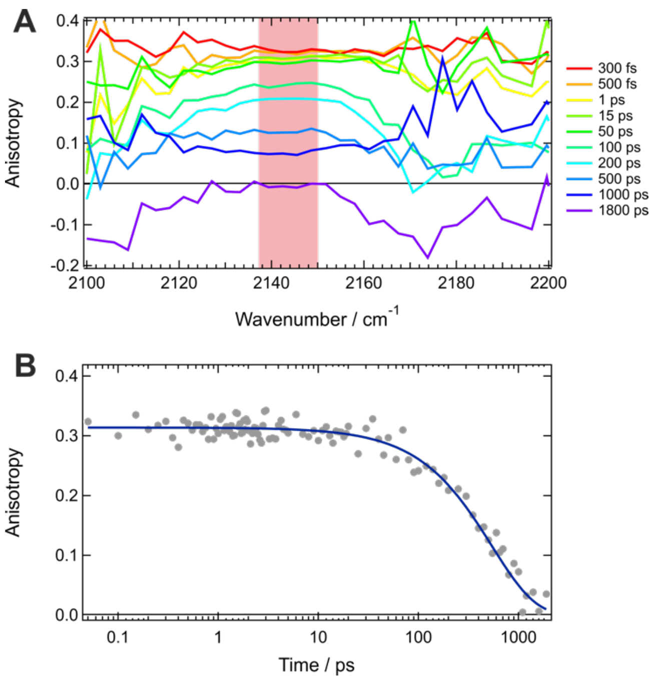

Figure S14. **(A)** Anisotropy of the TRIR signal in the C≡N stretch region measured with **ADA** in isopropanol and **(B)** temporal decay. The grey markers are experimental data points, while the solid line is the best monoexponential fit with a 540 ps time constant. The spectral range used for the analysis of the anisotropy values is highlighted in red in panel **A**.

5. Transient infrared measurements in HFP/$CHCl_3$ mixtures

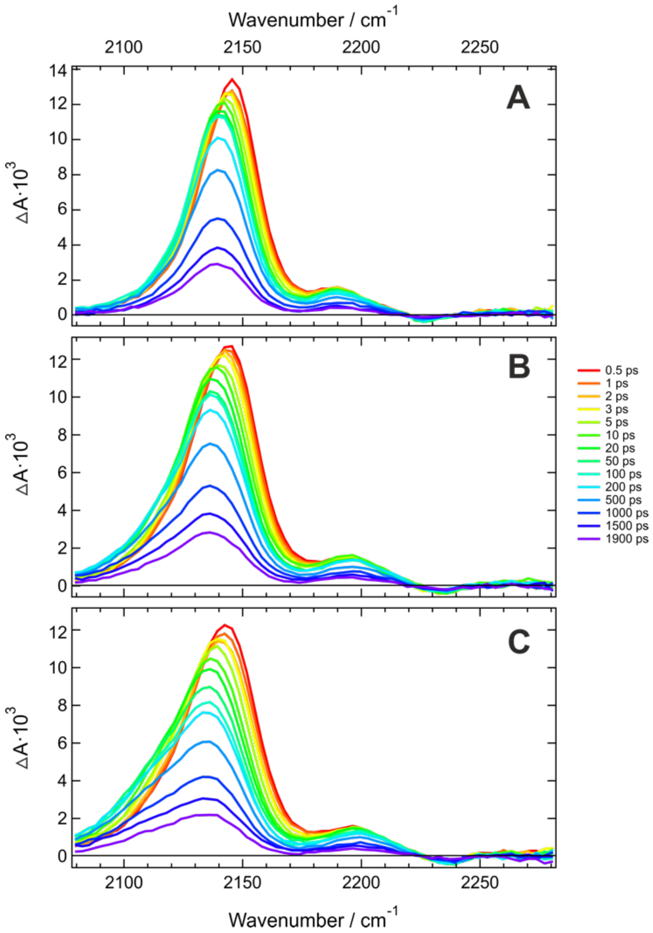

Figure S15. TRIR spectra in the C≡N stretching region measured at different time delays after excitation of **ADA** in $CHCl_3$ and various concentrations of HFP (0.1 M, **A**), (0.5 M, **B**), (1.0 M, **C**). Apart from the concentration of HFP, the experimental conditions were the same in all three cases and, thus, the intensities can be compared.

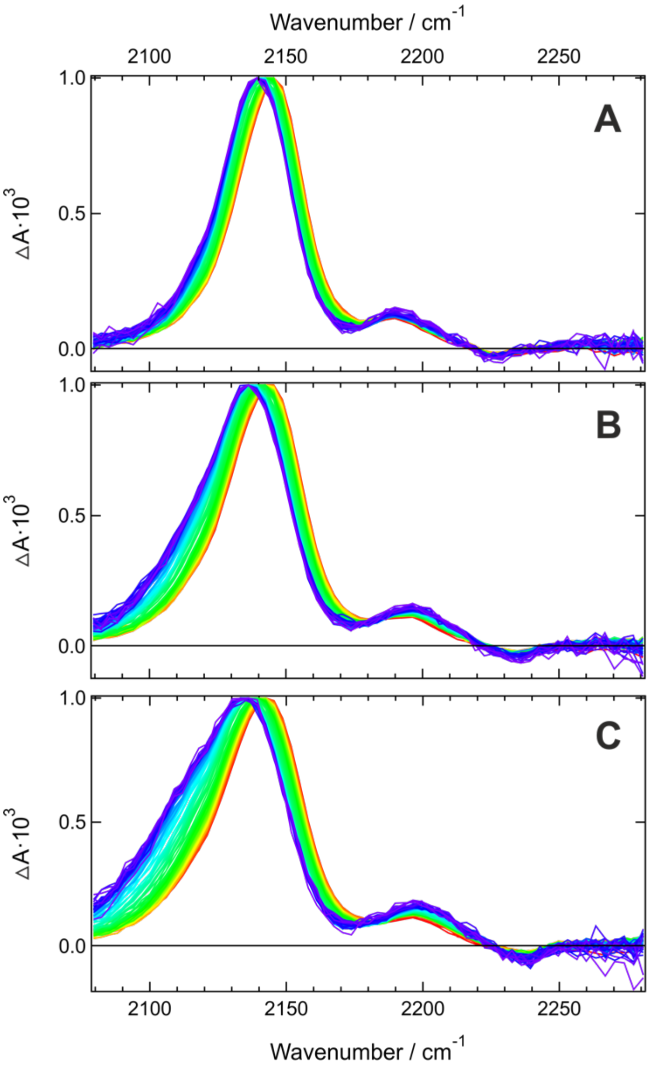

Figure S16. Same as Figure S15 after intensity-normalization of the TRIR spectra.

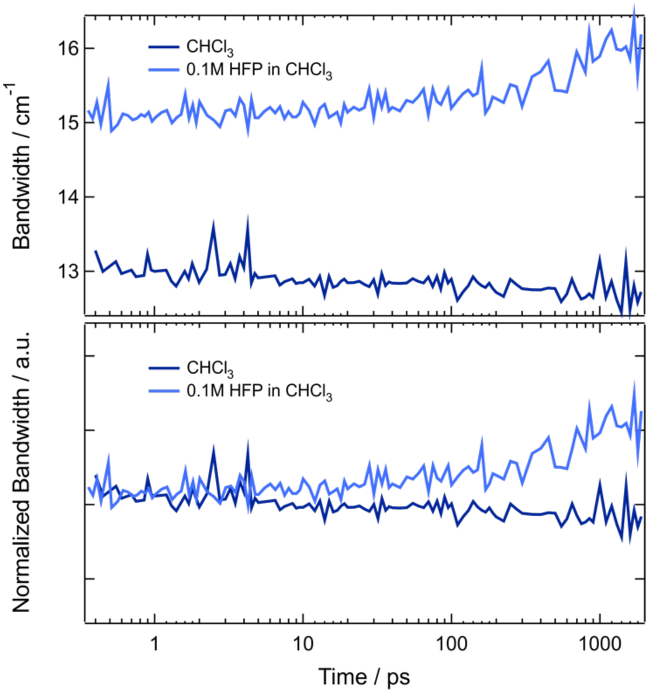

Figure S17. (Top) Time evolution of the bandwidth of the ESA1 C≡N band at ~2140 cm^{-1} extracted from the bandshape analysis of the TRIR spectra in chloroform and 0.1 M HFP (Figure S15A). (Bottom) Same as above after normalization. This figure clearly illustrates that, although the formation of the tight complex is not evident from the TRIR spectra at this HFP concentration, it nevertheless takes place, as testified by the band broadening. At 0.5 M and 1.0 M HFP, the appearance of a shoulder on the low-frequency side of the main band is visible in Figure S15B and C.

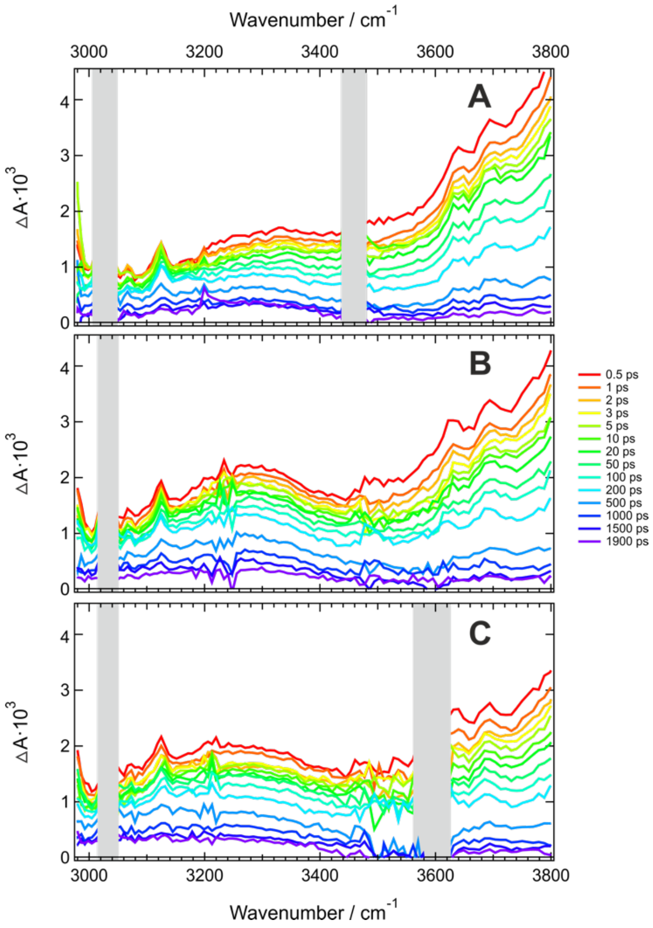

Figure S18. TRIR spectra recorded in the O-H stretching region at different time delays after excitation of **ADA** in $CHCl_3$ with 0.1 M (**A**), 0.5 M (**B**) or 1.0 M HFP. Contrary to the O-H band, the $S_2 \leftarrow S_1$ band is barely visible at long time delays. This apparently fast decay originates from the shift of the $S_2 \leftarrow S_1$ band upon formation of the tight H-bond complex, as discussed in the main text.

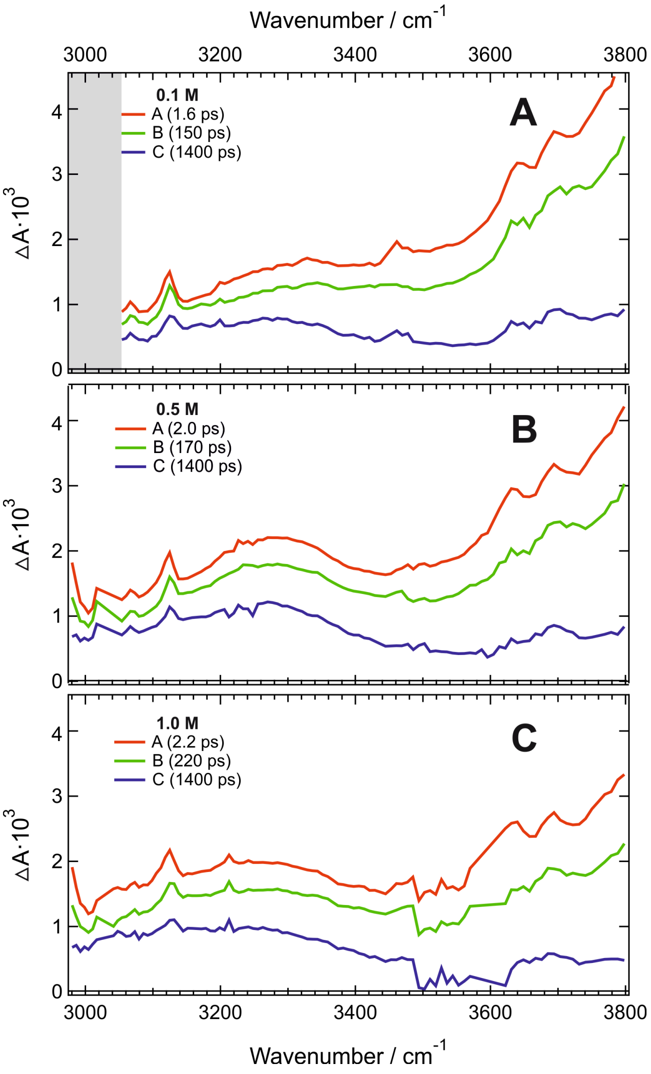

Figure S19. Evolution-associated difference spectra obtained from a global analysis of the transient IR absorption spectra recorded in the O-H stretching region at different time delays after 400 nm excitation of **ADA** in $CHCl_3$ with 0.1 (**A**), 0.5 (**B**), and 1.0 M (**C**) HFP assuming three successive steps.

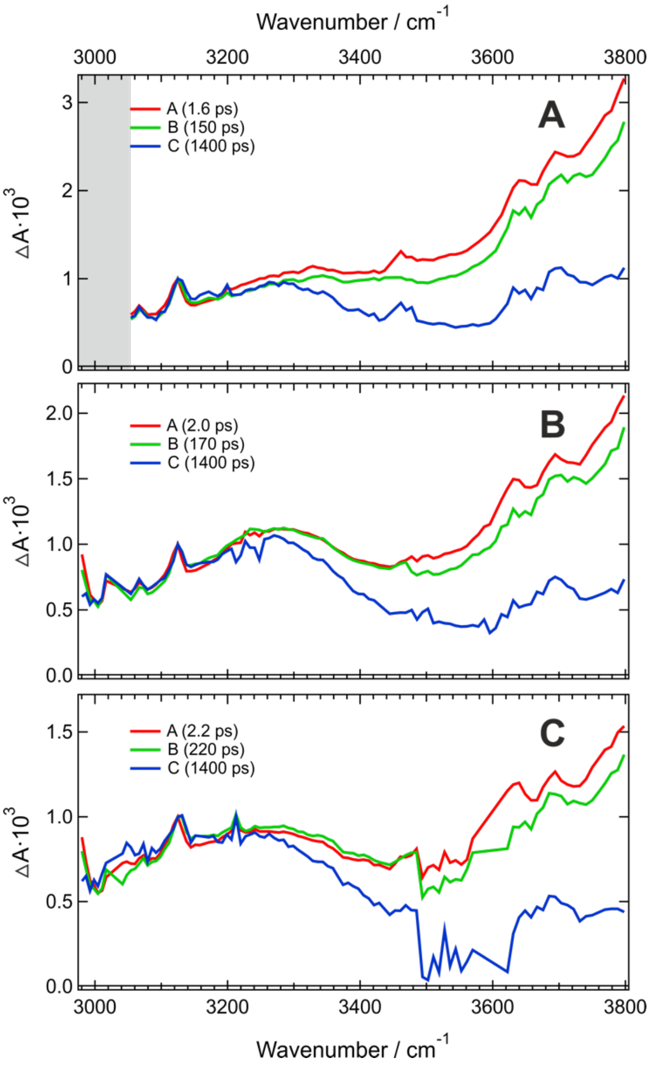

Figure S20. Same as in Figure S19 after normalization at the C-H stretch absorption peak. The strengthening of the H-bond with time can be seen as a redistribution of the band toward the lower frequencies.

6. References

(S1) Janiga, A.; Glodkowska-Mrowka, E.; Stoklosa, T.; Gryko, D. T. Synthesis and Optical Properties of Tetraaryl-1,4-Dihydropyrrolo[3,2-B]Pyrroles *Asian J. Org. Chem.* **2013**, *2*, 411-415.

(S2) Koch, M.; Letrun, R.; Vauthey, E. Exciplex Formation in Bimolecular Photoinduced Electron-Transfer Investigated by Ultrafast Time-Resolved Infrared Spectroscopy *J. Am. Chem. Soc.* **2014**, *136*, 4066-4074.

(S3) Bredenbeck, J.; Hamm, P. Versatile Small Volume Closed-Cycle Flow Cell System for Transient Spectroscopy at High Repetition Rates *Rev. Sci. Instrum.* **2003**, *74*, 3188-3189.

(S4) Dereka, B.; Rosspeintner, A.; Krzeszewski, M.; Gryko, D. T.; Vauthey, E. Symmetry-Breaking Charge Transfer and Hydrogen Bonding: Toward Asymmetrical Photochemistry *Angew. Chem. Int. Ed.* **2016**, *55*, 15624-15628.

(S5) Lewis, J. E.; Maroncelli, M. On the (Uninteresting) Dependence of the Absorption and Emission Transition Moments of Coumarin 153 on Solvent *Chem. Phys. Lett.* **1998**, *282*, 197-203.
